# *MIR396-GRF/GIF* enhances *in planta* shoot regeneration of *Dendrobium catenatum*

**DOI:** 10.1186/s12864-024-10360-9

**Published:** 2024-05-31

**Authors:** Zhenyu Yang, Meili Zhao, Xiaojie Zhang, Lili Gu, Jian Li, Feng Ming, Meina Wang, Zhicai Wang

**Affiliations:** 1 Shenzhen Key Laboratory for Orchid Conservation and Utilization, the National Orchid Conservation Center of China and the Orchid Conservation & Research Center of Shenzhen, Shenzhen, 518114 China; 2 Key Laboratory of National Forestry and Grassland Administration for Orchid Conservation and Utilization, the National Orchid Conservation Center of China and the Orchid Conservation & Research Center of Shenzhen, Shenzhen, 518114 China; 3 Shanghai Key Laboratory of Plant Molecular Sciences, College of Life Sciences, Shanghai Normal University, Shanghai, 200234 China; 4 Xinjiang Key Laboratory of Grassland Resources and Ecology, College of Grassland Sciences, Xinjiang Agricultural University, Urumqi, 830052 China

**Keywords:** Orchid, Monocotyledonous species, Plant growth, Transformation

## Abstract

Recent studies on co-transformation of the growth regulator, *TaGRF4-GIF1* chimera (*Growth Regulating Factor 4-GRF Interacting Factor 1*), in cultivated wheat varieties (*Triticum aestivum*), showed improved regeneration efficiency, marking a significant breakthrough. Here, a simple and reproducible protocol using the GRF4-GIF1 chimera was established and tested in the medicinal orchid *Dendrobium catenatum*, a monocot orchid species. *TaGRF4-GIF1* from *T. aestivum* and *DcGRF4-GIF1* from *D. catenatum* were reconstructed, with the chimeras significantly enhancing the regeneration efficiency of *D. catenatum* through *in planta* transformation. Further, mutating the *microRNA396* (*miR396*) target sites in *TaGRF4* and *DcGRF4* improved regeneration efficiency. The target mimicry version of *miR396* (*MIM396*) not only boosted shoot regeneration but also enhanced plant growth. Our methods revealed a powerful tool for the enhanced regeneration and genetic transformation of *D. catenatum*.

**Supplementary Information:**

The online version contains supplementary material available at 10.1186/s12864-024-10360-9.

## Introduction

In the past few decades, plant transformation proved to be a basic tool for the characterization of gene functions and the development of genetically modified crops [1, 2]. Plant genetic transformation is highly species- and genotype-dependent, often causing bottlenecks in crop genetic engineering and gene editing. With *Agrobacterium tumefaciens*-mediated genetic transformation efficiency being low in hormone-based tissue culture of orchids, several transformation and regeneration systems were developed in various orchid species [3]. However, these methods were time-consuming and inefficient, resulting in the production of only a small number of plants. Another challenge in the genetic transformation of orchids was the extended juvenile phase of immature tissues [4], hindering the functional analyses of genes. Additionally, compared to dicots, monocot orchids are more difficult to transform with *A. tumefaciens* as dicots are the natural hosts of the pathogen [5]. Hence, effective transformation and regeneration methods were required for orchids to produce a large number of uniform seedlings within a short time.

The constitutive expression of a small class of transcription factors (TFs), including the GRF (Growth Regulating Factor) and its corresponding co-activator GIF (GRF Interacting Factor), significantly enhanced the transformation and regeneration of fertile plants with normal phenotypes [6, 7]. This rendered the conditional expression or removal of the transgenes unnecessary. GRFs are highly conserved plant-specific TFs that influence cell proliferation and size [8, 9], thereby regulating meristem formation and plant growth and development [10]. GRF proteins may interact with corresponding co-factor GIFs to create a functional transcriptional complex in vivo [8], with the activity of the complex being precisely regulated at multiple levels. Studies showed that microRNA396 (*miR396*) post-transcriptionally repressed most *GRF* members and guided *GRF* mRNAs for cleavage or transcriptional arrest, thereby controlling the *GRF*-*GIF*-dependent processes [8]. Mutating the *miR396* target sites in *GRF*s showed an increase in the *GRF* transcripts, with enhanced activities in the GRF-GIF complexes [11]. The *miR396-GRF/GIF* module was found to recruit Switch/Sucrose Nonfermenting (SWI/SNF) chromatin remodeling complexes to regulate the expression of target genes and determine the meristematic identity for organogenesis [8, 12]. For instance, the PpnGRF-GIF complex from poplar inhibited the expression of *Cytokinin Oxidase/Dehydrogenase 1* (*PpnCKX1*), leading to cytokinin accumulation and meristematic induction [13]. The GRF4-GIF1 fusion protein from wheat, citrus, and grape significantly enhanced transformation efficiency, yielding fertile transgenic plants with normal phenotypes in multiple plant species, including wheat (*Triticum aestivum*), rice (*Oryza sativa*), citrus (*Citrus sp.*), grape (*Vitis vinifera*), and hemp (*Cannabis sativa*) [7]. Introducing synonymous mutations to the *miR396* target site of TaGRF4 resulted in the mTaGRF4-GIF1 complex, outperforming the original TaGRF4-GIF1 complex [11]. The *ClGRF4-GIF1* chimera from *Citrullus lanatus* boosted the transformation efficiency of watermelon in a genotype-independent manner [1]. *AtGRF5* and its homologs from *Arabidopsis thaliana* enhanced shoot regeneration and transformation efficiency in maize (*Zea mays*), canola (*Brassica napus*), soybean (*Glycine max*), sugar beet (*Beta vulgaris*), and sunflower (*Helianthus annuus*) [6]. Superior performance in both monocots and dicots and the accelerated transformation process highlighted the broad application potential of the *GRF-GIF* chimera.

This study aimed to investigate the impact of the *GRF4-GIF1* chimera on the regeneration efficiency of juvenile explant tissues from the monocot species *D. catenatum*. Our findings demonstrated that a constitutive expression of the *GRF4-GIF1* chimera, its mutated variant *mGRF4-GIF1*, and the target mimicry version *MIM396* significantly enhanced shoot regeneration efficiencies in *D. catenatum*. Notably, the introduction *MIM396* into *D. catenatum* significantly enhanced plant growth. Compared to the traditional methods of orchid regeneration, our strategy proved to be efficient, with significant potential to expedite orchid research and molecular breeding in the future.

## Results

### Identification of DcGRF4 and DcGIF1, and the construction of the GRF4-GIF1 chimera

**Fig. 1:**
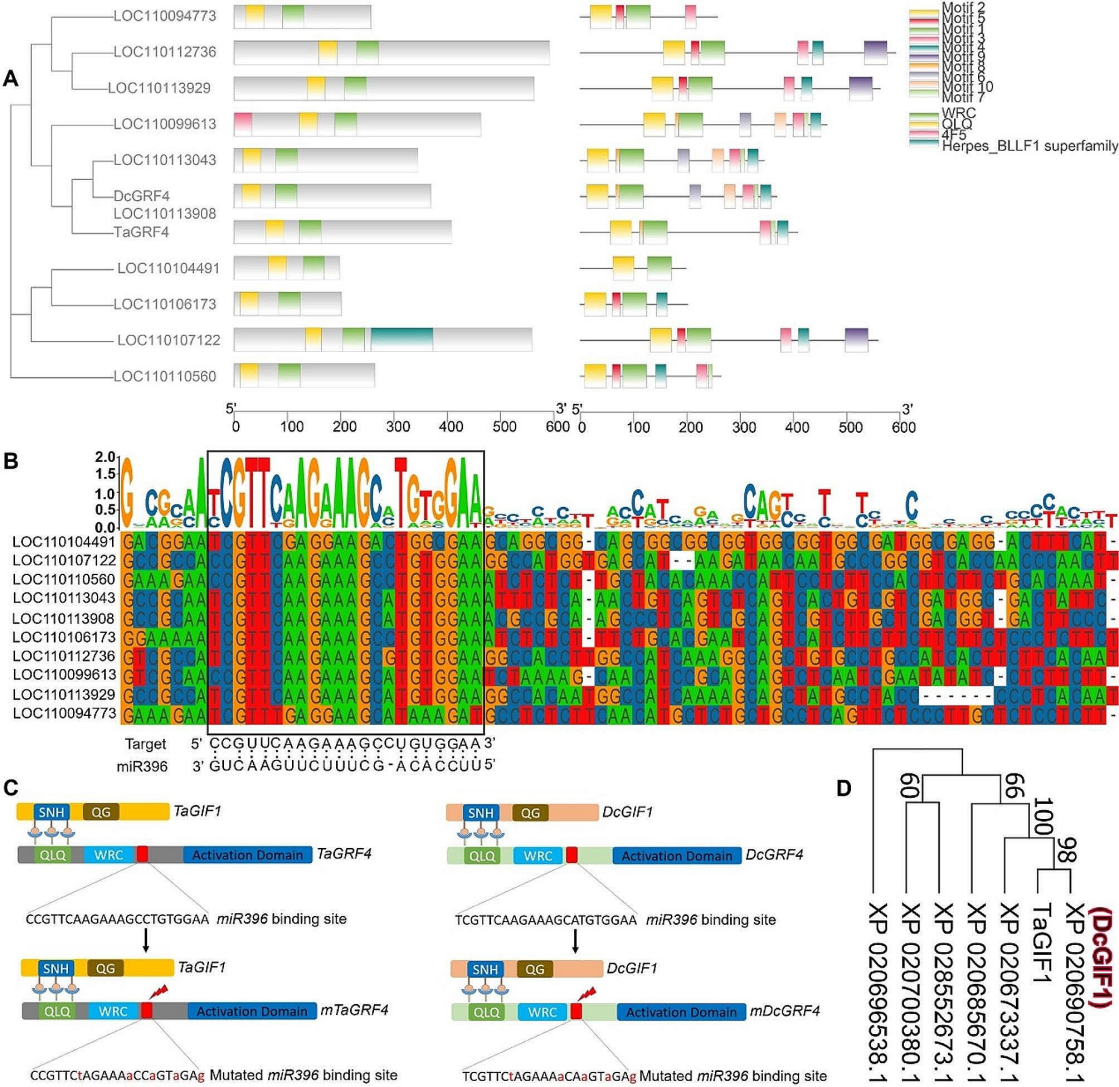
Phylogenetic analysis of DcGRFs. **(A)** Phylogenetic relationship between DcGRFs and TaGRF4. The phylogenetic tree was based on the sequence of the ten proteins bearing QLQ and WRC domains identified in *D. catenatum* and the TaGRF4 protein. The combined tree, conserved domains, and motifs were constructed using TBtools. **(B)** Comparison of the complementary site of *miR396* with the *DcGRF* genes is indicated by the black frame. **(C)** Schematic representation of *TaGRF4*, *TaGIF1*, mutated *TaGRF4* (left panel), *DcGRF4*, *DcGIF1*, and mutated *DcGRF4* (right panel). The interaction between SNH and QLQ domains is indicated. *mTaGRF4* and *mDcGRF4* were created by introducing five single-site mutations into the *miR396* target sites of *TaGRF4* and *DcGRF4*, respectively. **(D)** Phylogeny of DcGIFs from *D. catenatum*. Protein sequences were used for the analysis. TaGIF1 was used to identify the DcGIF1 homologue in *D. catenatum*

Based on recent reports demonstrating the regeneration-improving effects of the *GRF4-GIF* chimera in various plant species [7, 11], we speculated whether it could be applied to orchids having time-consuming and inefficient regeneration processes. To study this, the presence of *GRF*s in *D. catenatum* was initially examined, with ten homologues being identified using the profile Hidden Markov Model (HMM) and phylogenetic analysis (Fig. 1A). Of them, *DcGRF4* (LOC110113908) was designated to have the highest homology to *TaGRF4*, a GRF transcription factor gene from *T. aestivum* [7]. Additionally, eight of the ten *DcGRF*s were predicted to be targeted by *miR396*, except for LOC110104491 and LOC110094773 (Fig. 1B). To create *mDcGRF4*, five mutations were introduced into the *miR396* binding site of *DcGRF4* (Fig. 1C), following the corresponding sequence in *mTaGRF4*. To create the DcGRF4-GIF1 chimera, a phylogenetic tree was constructed to identify the closest homologue of TaGIF1, designated as DcGIF1 (Fig. 1D). There were six members of DcGIFs in the *D. catenatum* genome.

### *GRF4-GIF1* chimera enhances *in planta* shoot regeneration of *D. catenatum*

**Fig. 2:**
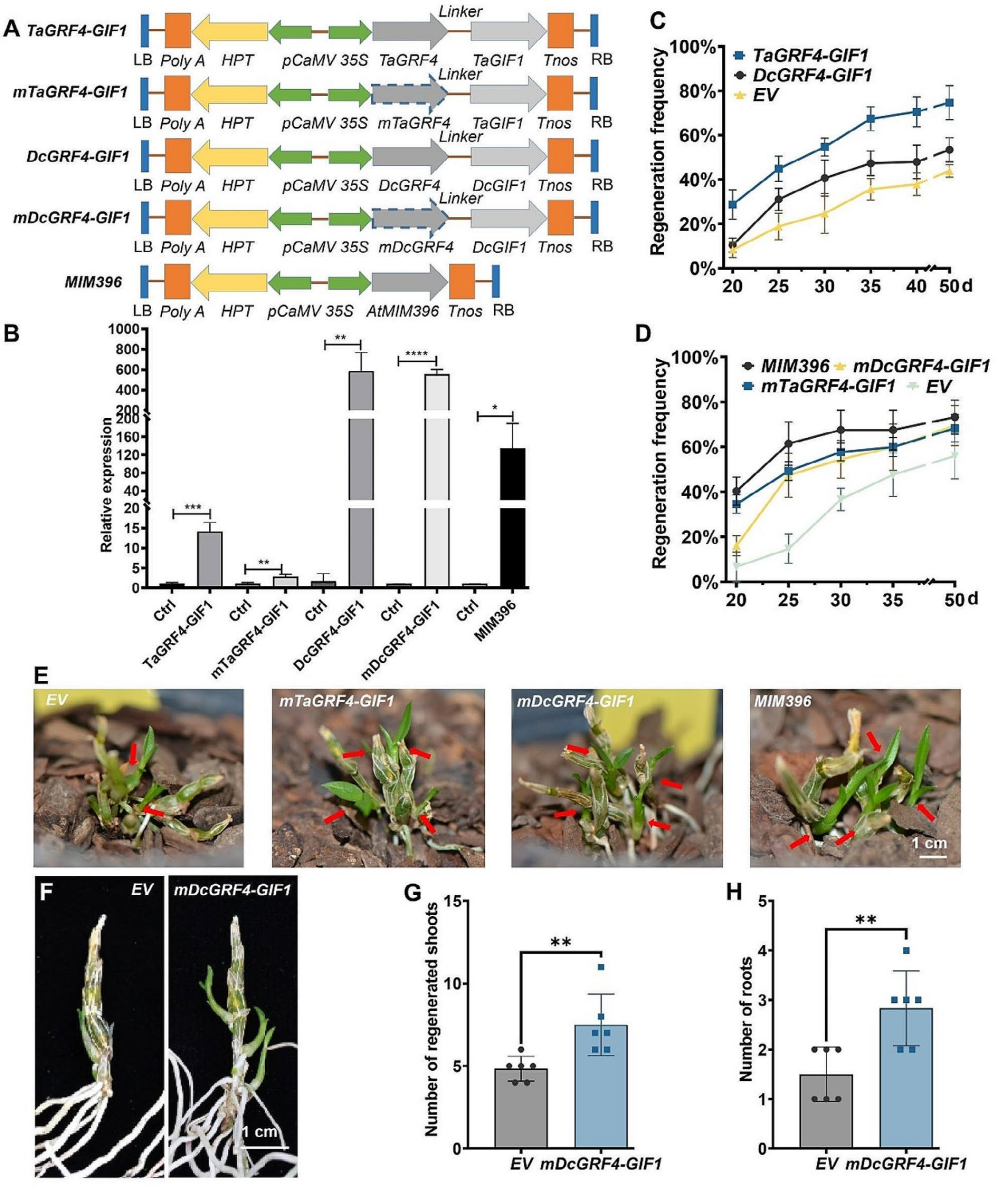
The *GRF4-GIF1* chimera enhanced *in planta* regeneration of *D. catenatum*. **(A)** Diagrams illustrating T-DNAs used, including the left border (LB), *poly A* tail (*Poly A*), *hptII*, *35 S CaMV* promoter (*p35S*), and the right border (RB). Elements in the vector backbone were not shown. **(B)** qRT-PCR verification of *GRF4-GIF1* and *MIM396* expression in respective constructs. Ctrl means empty vector control. The bar graph shows the relative levels of transcripts with respect to the internal control gene *Actin7*. Each bar represents mean ± SD (*n* = 3). **(C)** Cumulative changes indicate that *GRF4-GIF1* promoted shoot regeneration. **(D)** Cumulative changes demonstrate that both *mGRF4-GIF1* and *MIM396* enhanced shoot regeneration. Regeneration frequency = regenerated shoots/total plantlets × 100%. At least three replicates with five plantlets in each were included for each construct. **(E)** Representative images display improved shoot regeneration and growth by *mGRF4-GIF1* and *MIM396*. **(F)** Shoot regeneration of *in planta*-infiltrated plantlets on SIM in sealed bottles. **(G-H)** Statistical analysis of regenerated shoots **(G)** and total roots **(H)** from *in planta*-infiltrated plantlets in sealed bottles (*n* ≥ 5). At least five replicates with five plantlets in each were included for each construct. The scale bar represents 1 cm

To evaluate the robustness of the *GRF-GIF* chimera in monocot orchids, we transformed *in planta TaGRF4-GIF1* from *T. aestivum, DcGRF4-GIF1* from *D. catenatum*, mutated versions of *GRF4-GIF1* (*mTaGRF4-GIF1* and *mDcGRF4-GIF1*), as well as a *miR396* target-mimicry *MIM396* vector (Fig. 2A), into the *D. catenatum* stem nodes. Tissue-culture-derived nine-month-old *D. catenatum* seedlings were pruned to remove all visible shoot meristems. The cut sites and stem nodes were perfused with *Agrobacterium* solutions containing either an empty vector or a vector with the *GRF4-GIF1* chimera. The infected seedlings were grown in bark pots. The expression of the constructs used was verified by qRT-PCR (quantitative real time polymerase chain reaction) analysis, demonstrating enhanced expression of the target genes (Fig. 2B). Groups infiltrated with the *GRF4-GIF1* chimeras showed increased shoot formation compared to the vector control (Fig. 2C). Similarly, *mTaGRF4-GIF1* and *mDcGRF4-GIF1* also increased shoot regeneration efficiency, with *MIM396* further boosting the efficiency (Fig. 2D). This resulted in faster growth of the regenerated plantlets compared to the vector control (Fig. 2E). When *mDcGRF4-GIF1* infiltrated plantlets were transferred to the shoot induction medium (SIM) in sealed bottles, an increase in the number of shoots and roots was observed (Fig. 2F-H). The stable moisture and rich nutrients in sealed bottles likely facilitated the initiation of shoots and roots.

**Fig. 3:**
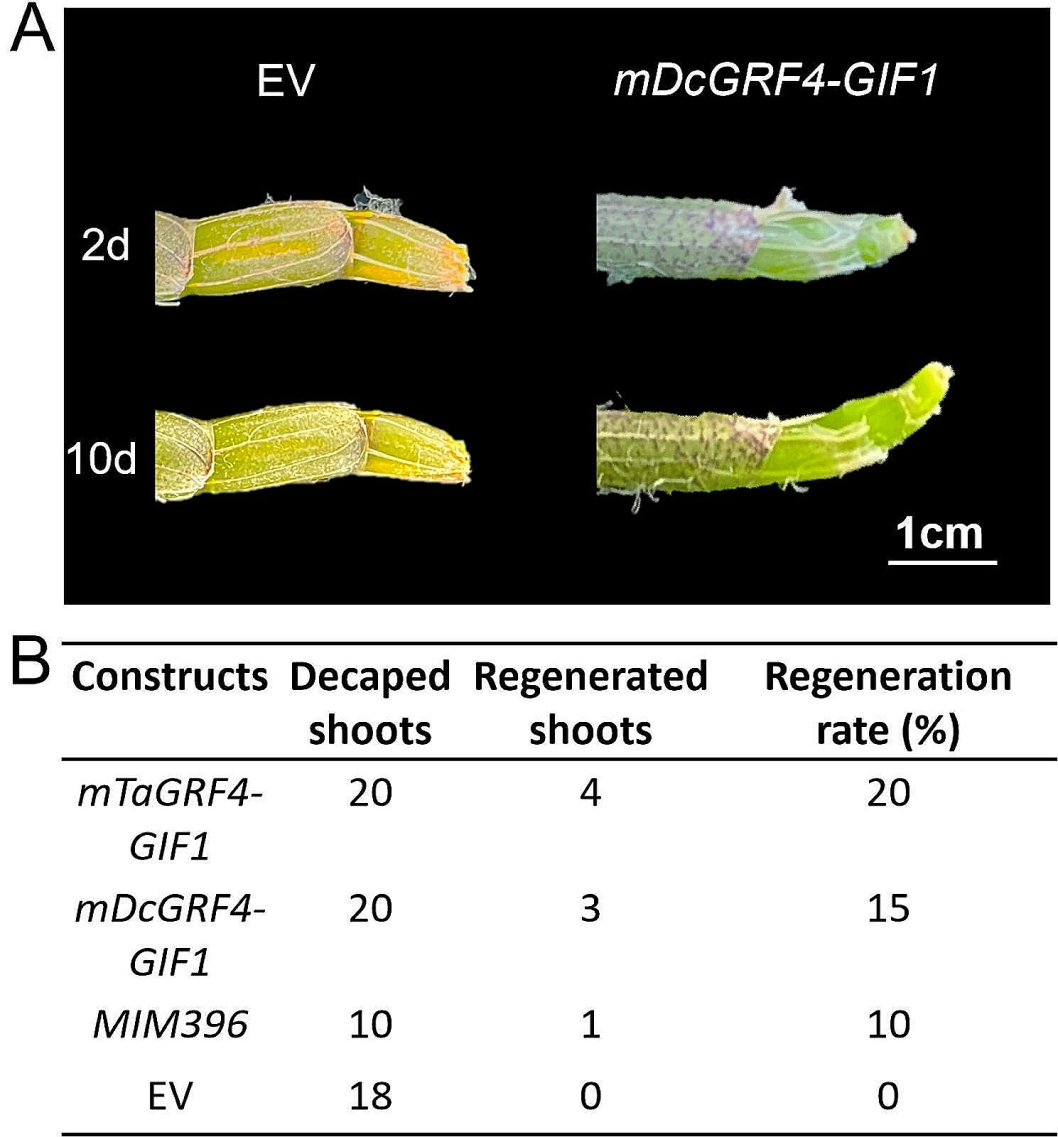
*In planta* transformation of GRF4-GIF1 improved shoot regeneration of adult plants. Three-year-old adult plants of *D. catenatum* were decapped and *Agrobacterium* carring *GRF4-GIF1* or EV constructs were injected *in planta* at the cut sites. **(A)** Representative images of regenerated shoots were shown. **(B)** Shoot regeneration rates were recorded three weeks after injection. Bar in **(A)** represents 1 cm

To further demonstrate its effectiveness, the *GRF4-GIF1* chimera was applied to three-year-old adult plants of *D. catenatum*. Clear protrusion (2 dpi) and elongation (10 dpi) of apical shoots were observed from the cut sites injected with *mDcGRF4-GIF1*, while those injected with an empty vector remained unchanged (Fig. 3A). Three weeks after injection, the shoot regeneration rate for *mTaGRF4-GIF1* transformants reached 20%, followed by *mDcGRF4-GIF1* (15%), and *MIM396* transformants (10%). However, no protrusion or elongation was observed at the cut sites of empty vector transformants (Fig. 3B). Thus, the *GRF4-GIF1* chimera not only improved the shoot regeneration efficiency of young seedlings but also enhanced shooting in the adult plants of *D. catenatum*, reconfirming the robustness of this technology in *D. catenatum*.

### *GRF4-GIF1* chimera boosts transformation in *D. catenatum*

**Fig. 4:**
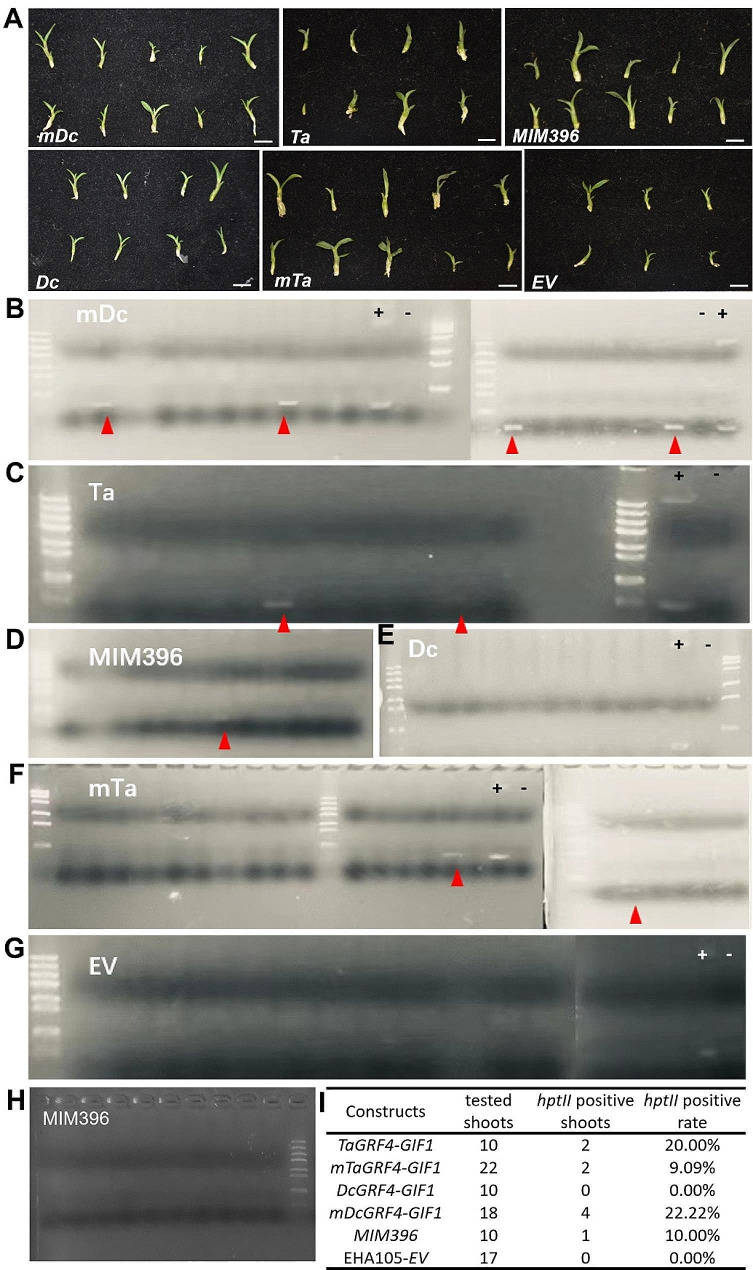
Genomic PCR identification of transgenes. **(A)** Representative images of regenerated shoots used for transgene detection. **(B-G)** Genomic PCR detection of regenerated shoots for *hptII* transgenes. “+” indicates the plasmid of pNC-Cam1304-*35 S* used as a positive control. “-” indicates genomic DNA from wild-type plants used as a negative control. The target bands were marked with underlined red triangles. **(H)** Amplification of *nptII* in *MIM396* transformants to avoid bacterial contamination. **(I)***In planta* transformation efficiencies using *GRF4-GIF1*-based technology. The transformation efficiency of *hptII* was calculated by dividing the number of *hptII* positive shoots by the total number of shoots tested. The bars in the graph represent 1 cm

All plasmid vectors used in this study contained the *hptII* (*hygromycin phosphotransferase II*) gene. To confirm the presence of transgenes in the genome of independently regenerated shoots, genomic PCR was performed (Fig. 4A). Of the 60 shoots regenerated from *GRF4-GIF1* and *MIM396* of *in planta*-transformed *D. catenatum* seedlings, nine were found to be *hptII* positive, accounting for 15.0% (Fig. 4B-G). To avoid contamination, *nptII* (*neomycin phosphotransferase II*) in the plasmid backbone was amplified in *MIM396* transformants (Fig. 4H). Notably, *mDcGRF4-GIF1* displayed the highest transformation efficiency of 22.2%, while none of the shoots from the vector control showed positive results (Fig. 4I). These results indicated that the *GRF4-GIF1* chimera not only enhanced shoot regeneration but also increased the *in planta* transformation efficiency of *D. catenatum*.

### *MIM396* promotes *D.* *catenatum* growth

**Fig. 5:**
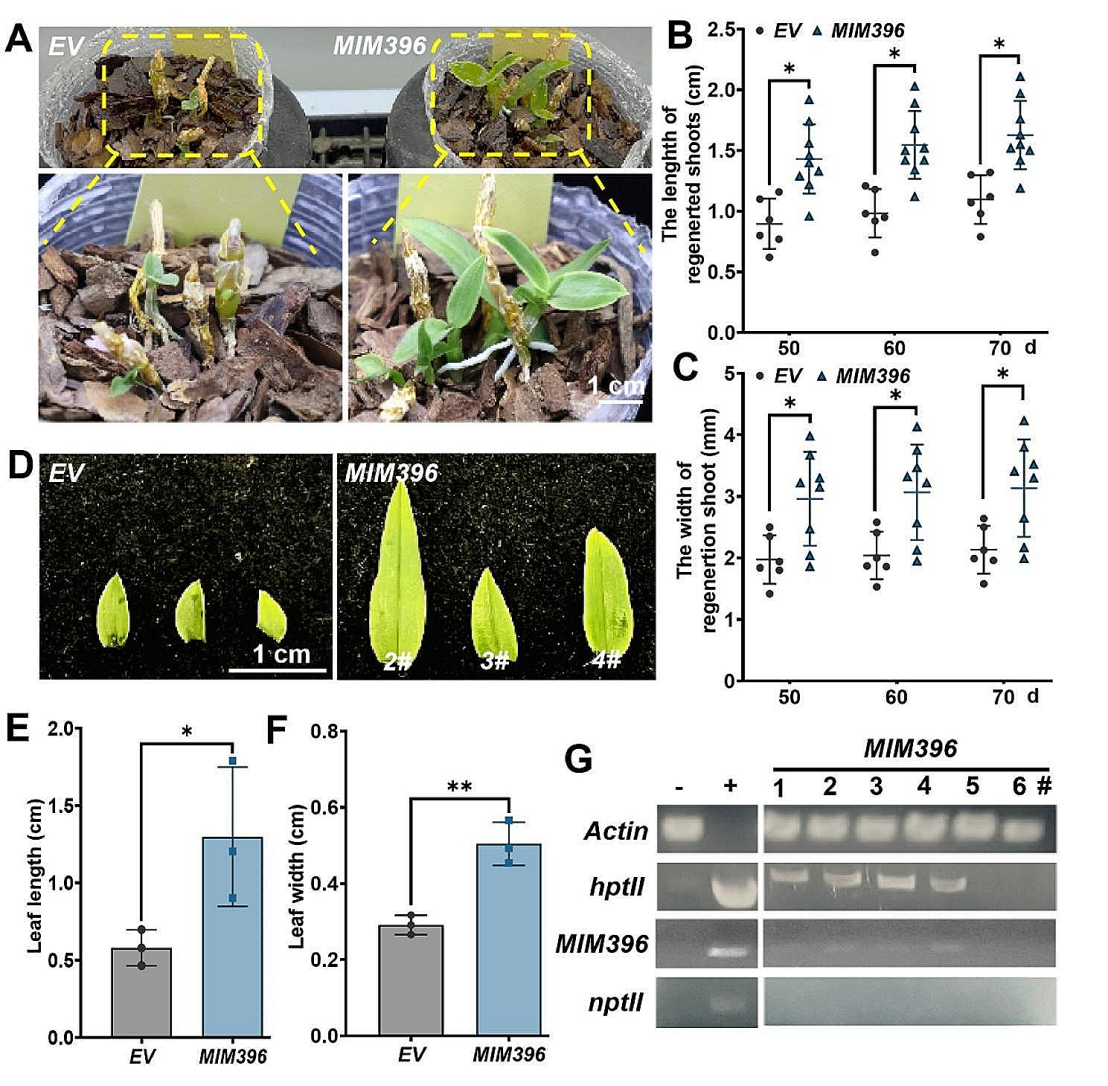
*MIM396* enhanced plant growth and leaf development in *D. catenatum*. **(A)** Representative images showing enhanced plant growth by *MIM396*, with zoomed-in details given below. **(B)** Statistical analysis of the length of the regenerated shoots. **(C)** Statistical analysis of the width of the regenerated shoots. **(D)** Representative images comparing the leaves of *MIM396* with those of vector control transgenic plantlets. **(E-F)** Statistical analysis of leaf length **(E)** and leaf width **(F)** for *MIM396* or vector control transformants. **(G)** Genomic PCR verification of *MIM396* in transgenic *D. catenatum* plantlets. To avoid contamination, *nptII* in the plasmid backbone was amplified. “-” indicates wild-type plants while “+” represents the *MIM396* plasmid used as a positive control. The scale bar represents 1 cm

Further studies were made using *in planta*-transformed transgenic plants to verify that *MIM396* improved plant growth. One-month-old *MIM396* transgenic plants exhibited robust growth (Fig. 5A), with higher plant height (Fig. 5B) and larger stem diameter compared to the vector control (Fig. 5C). Moreover, the leaves of *MIM396* transgenic plants were larger (Fig. 5D), with longer leaf length (Fig. 5E) and broader leaf width (Fig. 5F). The presence of *MIM396* and *hptII* transgenes was identified in plants with strong phenotypes but not in wild-type plants or plants without phenotypes (Fig. 5G). These results strongly demonstrated that *miR396* regulated not only shoot regeneration but also plant growth and leaf development in *D. catenatum*.

## Discussion

*Agrobacterium* infects a wide range of plant species, with host restrictions that are generally more efficient in dicots than in monocots [14]. Despite advancements in transformation technology, achieving successful genetic transformation in certain plant species remains a major challenge [15]. To overcome genotype dependence partially and improve the efficiencies of plant transformation and regeneration, the introduction of developmental genes (*DG*s) in both monocots and dicots was found effective. This was demonstrated by the combined use of *GRF* and *GIF* genes [7, 11, 16]. Over the past four decades, pioneering studies identified *DG*s to play a crucial role in growth and development. Overexpression of *DG*s showed significant improvements in somatic embryogenesis and shoot regeneration as well as transformation frequency, along with a reduction in the duration of the transformation process and the expansion of transformable genotypes [17, 18]. Some *DG*s encode transcription factors (TFs), including WUSCHEL (WUS), which maintains pluripotent stem cell identity in shoot and flower meristems [19] and BABY BOOM (BBM), which controls embryo identity [20]. Functions of WUS and BBM orthologs are widely conserved across different plant species, including dicots and monocots. By ectopically expressing WUS orthologs or combining WUS with BBM from various species, efficient transformation and regeneration were achieved in different genotypes, including in *Z. mays* [21], *O. sativa* [22], *Populus tomentosa* [23], *Coffea canephora* [24], *Gossypium hirsutum* [25], *Capsicum chinense* [26], *A. thaliana* [27], *Nicotiana tabacum* [28], and *Sorghum bicolor* [29]. Another strategy was to use different combinations of *DG*s to induce *de novo* meristems in dicots without tissue culture [30]. However, when *DG*s, including *WUS* and *BBM*, were constitutively expressed, negative pleiotropic effects were observed on further development, including disorganized shoots and floral meristems [27], infertility, and shoot necrosis [21]. Hence, it was necessary to carefully control their expression strength and remove them from the engineered plants [31, 32]. Consequently, there was a need for new transformation methods with high efficiency, ease of use, and less genotype-dependent characteristics. *DG*s-based transformation reduced genotype dependence, with improved efficiency and speed. The *miR396*-*GRF*/*GIF* signaling module recently emerged as an alternative to *BBM* and *WUS* to enhance plant regeneration capacity and transformation efficiency [7, 11]. *TaGRF4* and *TaGIF1* were fused in a chimera, with the forced proximity increasing the chimera’s ability to induce regeneration [7]. By mutating the *miR396* target sites in *TaGRF4* within the *TaGRF4-GIF1* complex, regeneration efficiency was improved further [7, 11].

Based on the previous works, our study utilized the Ta/mTaGRF4-GIF1 fusion protein with additional modifications. First, *TaGRF4* and *TaGIF1* homologs were cloned and verified in *D. catenatum* to enhance shoot regeneration. Second, *MIM396*, the *miR396* target mimicry version, was tested. Our results showed that overexpression of *GRF4-GIF1* and *MIM396* significantly increased shoot regeneration efficiency of *D. catenatum*. The method developed for *in planta D. catenatum* transformation showed potential for research and molecular breeding. With GRF-GIF chimeras being plant-specific TF complexes and highly conserved in all land and charophyte plants [8], the GRF4-GIF1 chimera-based technology can be readily extended to other orchid species having low regeneration efficiencies.

In *Arabidopsis*, rice, and maize, the GRF-GIF chimera interacted with the SWI/SNF chromatin remodeler to repress the Polycomb Repressive Compex 2 (PRC2) in the transcription of developmental regulators [32]. This consequently triggered cell proliferation in a wide range of organs [6]. However, the role of the *GRF* genes in somatic embryogenesis is still poorly understood [33], with further research being required to uncover the functional mechanisms of the *GRF* genes in the transcriptional regulation of key meristematic or embryonic regulators. Additionally, *MIM396* transgenic *D. catenatum* was observed to exhibit enhanced plant growth and leaf development, eliminating the need for laborious and time-consuming removal or inactivation of transgenes. Similar functions of *miR396* were also well-documented in rice and *Arabidopsis* [34, 35]. However, the beneficial growth-promoting effect of *D. catenatum* in breeding and commercial production might not be suitable for developmental biology research.

Though this technology demonstrated robustnees in *D. catenatum*, it cannot be assumed to have equal success in the given orchid species. Variations among genotypes could still exist, with different success rates for different constructs used. However, as GRFs are widely distributed and highly conserved among plant species, it could be worthwhile to examine the activity of all GRFs in a given species to determine its most effective member in boosting regeneration and organogenesis. Hence, in certain cases, including plants deemed recalcitrant, an optimized specific set of GRFs, either individually or in combination, could yield better results. For instance, when heterologously expressed in canola, *Arabidopsis AtGRF9* performed better in plant regeneration compared to *AtGRF5* [6]. *AtGRF5* exhibited no activity in maize and was less effective compared to endogenous *GRF5* homologues in soybean and sunflower [32]. Alternatively, to overcome the specificity of the GRF-GIF chimera, a synthetic complex can be created containing only the conserved functional domains [32]. These methods can be tested in *D.catenatum* and other orchid species in the future.

To bypass the tedious in vitro tissue culture regeneration, alternative *in planta* approaches were developed to introduce transgenes directly into intact plant tissues of various species, including *Arabidopsis*, rice, wheat, and maize [36, 37]. This approach took advantage of natural biological processes to regenerate transgenic plants, simplifying the procedure and reducing its dependence on genotype [31]. Here, we developed a method for *in planta* transformation of *D. catenatum* using *Agrobacterium*-mediated delivery of the *GRF4-GIF1* chimera. The method utilized the meristematic tissues of young seedlings, without the need for embryogenic callus induction, regeneration, or antibiotic selection. Vegetative meristems were targeted for *in planta* transformation, as demonstrated in studies using snapdragons [38], sweet potato [39], and sugarcane [40], where the formation of chimera was not a major concern.

## Conclusion

The present study provides a straightforward and replicable protocol for higher regeneration efficiency of *D. catenatum*. The expression of the *GRF4-GIF1* chimera, *mGRF4-GIF1*, and *MIM396* improved shoot regeneration efficiency through *in planta* transformation. The performance of the *GRF4-GIF1* chimera in *D. catenatum* demonstrated its wide-range potential to enhance the plant regeneration efficiency of various orchid species in the future.

## Materials and methods

### Plant materials

Monocot orchid species, *Dendrobium catenatum* was used in this study. The source plants of *Dendrobium catenatum* were collected from Guangdong province, P.R. China, in 2012. They were authenticated by Professor Zhongjian Liu from the National Orchid Conservation Center of China and a voucher specimen (Z.J.Liu6870) has been deposited there. Seeds of *Dendrobium catenatum* were collected and stored at our orchid conservation center (China National Orchid Conservation Center, Shenzhen, China). Potted *Dendrobium catenatum* plants were cultured in either sphagnum moss or bark medium and maintained in a greenhouse under natural light conditions at temperatures ranging from 25 to 28℃.

### Bacterial strains and binary vector

For cloning, *DH5α* competent cells from TransGen (CD501-03, Beijing, China) and ccdB survival^[TM]^ [2] cells *DB3.1* from Biomed (BC111-01, Beijing, China) were used. For transformation, hypervirulent disarmed *Agrobacterium* strains, EHA105 (BC307-01) was used. The *TaGRF4-GIF1* fragment was chemically synthesized and cloned into the pNC-Cam1304-*35 S* vector from NC Biotech (Hainan, China) under a *35 S Cauliflower mosaic virus* (*35 S*) promoter. *Agrobacterium* was electroporated following the standard protocol [37] and grown on solidified or liquid Luria Broth (LB) medium supplemented with appropriate antibiotics (rifampicin, 25 mg/L; spectinomycin, 50 mg/L; kanamycin, 50 mg/L; Sangon Biotech, Shanghai, China) at 28℃. For plant transformation, *Agrobacterium* was streaked from glycerol stocks on LB media with appropriate antibiotics. Growing colonies were picked and inoculated in freshly prepared liquid LB media, in which the bacterium was grown overnight in the dark at 28℃. Suspension cultures were adjusted to OD_600_ = 1.0 with 200 µM acetosyringone (D134406, Sigma, Germany).

### Vector construction

Gateway^TM^-compatible destination vector pNC-Cam1304-*35 S*, containing the target gene cloning cassette controlled by a double *CaMV 35 S* promoter (2 × *p35S*) [41] was used. The resulting expression vector was electroporated into *Agrobacterium* for plant transformation. Primers used in this study are listed in the supplementary Table S1. Endogenous *D. catenatum* genes *DcGRF4* (LOC110113908) and *DcGIF1* (LOC110105545) were linked by a 4 × Ala linker and cloned into pNC-Cam1304-*35 S* with a hygromycin selectable marker, resulting in the final construct pNC-Cam1304-*35 S*-*DcGRF4-GIF1*. Mutated *GRF4* within *miR396*-targeting sites was similarly fused with *GIF1*. The coding regions of *TaGRF4-GIF1*, *mTaGRF4-GIF1*^11^, and *MIM396* [42] were chemically synthesized by the Beijing Genomics Institute (BGI, Beijing, China) and PCR-amplified using corresponding primers.

### *In planta* transformation

A single colony of *Agrobacterium* carrying *Ta/DcGRF4-GIF1*, *mTa/mDcGRF4-GIF1*, and *MIM396* was inoculated into 10.0 mL LB liquid medium with 50.0 mg/L kanamycin and 25.0 mg/L rifampicin. The cultures were incubated overnight in an orbital shaker with 180 rpm at 28℃. The *Agrobacterium* cultures were multiplied by diluting 10% of the bacterial culture with 40 mL liquid LB supplemented with antibiotics and continued to grow. When the *Agrobacterium* cultures reached an OD_600_ of 1.0, the cells were harvested by centrifuging at 6000 rpm for 10 min. The resulting pellets were resuspended in 40 mL of fresh liquid Murashige & Skoog medium supplemented with 5.0% sucrose, 0.1% Silwet L-77, and 50.0 mg/L acetosyringone for injection. Tissue-culture-derived nine-month-old *D. catenatum* seedlings or three-year-old adults were used as starting materials. Plants were decaped and the cutting sites and the stem nodes were inoculated with the *Agrobacterium* suspension carrying the *GRF4-GIF1* chimera and *MIM396* constructs using a syringe. Excess *Agrobacterium* suspension was removed by air drying on a Whatman No. 1 filter paper. The infected plantlets were then planted in bark pots covered with plastic film to maintain moisture for two days. New shoots regenerated approximately two weeks after injection.

### Genomic PCR

Genomic DNA (gDNA) was extracted for PCR analysis of the transformed plants. Approximately 100.0 mg of tissue from the regenerated shoots was collected and ground using liquid nitrogen. gDNA was isolated from the crushed, frozen materials using the Genomic DNA Purification kit (DP350-03, Tiangen, Beijing, China). PCR amplification of the 400-bp fragment of the *hptII* gene from the gDNA samples was performed using the 2×EasyTaq® PCR SuperMix (AS111-12, TansGen, Beijing, China). To avoid contamination of the bacterium, a 200-bp fragment of *nptII* was amplified. Equal loading was controlled through the amplification of a 200-bp fragment of *DcActin-7* (LOC104111011). The resulting PCR products were separated by electrophoresis on 2.0% (w/v) agarose gels. To calculate the transformation efficiency of *hptII*, the number of *hptII* positive shoots was divided by the total number of the tested shoots.

### RNA extraction and qRT-PCR analysis

To confirm the expression of the constructs, total RNA was extracted from the infiltrated one-month-old *N. tobacum* leaves using the Quick RNA isolation Kit (0416-50gk, Huayueyang, Beijing, China) following the manufacturer’s instructions. First-strand cDNA was synthesized from 1.0 µg of total RNA using the PrimeScript™ RT reagent Kit with gDNA Eraser (RR047B, Takara, Dalian, China) following the manufacturer’s instructions. qRT-PCR was performed using the Green qPCR MasterMix (MT521-03, Biomed, Beijing, China) in an ABI PRISM 7500 Fluorescent Quantitative PCR System (Thermo Fisher Scientific, Singapore). *NtActin7* (X63603.1) was used as the reference gene for internal control. Each sample was analyzed in triplicates and the 2^−ΔΔCt^ method was used to determine the relative gene expression.

### Statistical analysis

Regeneration frequency data was collected for each construct and means and standard deviation (SD) values were determined. All data was organized and formatted using the GraphPadPrism8 software package (La Jolla, CA, USA). Significance of difference between treatments and controls was assessed using an unpaired Student’s t-test, with a significance level of *p* < 0.05. All experiments followed a completely randomized design, with three to ten replicates per treatment.

### Electronic supplementary material

Below is the link to the electronic supplementary material.

Supplementary Material 1

## Data Availability

The authors confirm that all data from this study are available and can be found in this article and in Supplementary Information.

## Declarations

### Declarations

The study is conducted with plant materials that comply with relevant institutional, national, and international guidelines and legislation.
