# Supplementary material for: MIR396-GRF/GIF enhances in planta shoot regeneration of Dendrobium catenatum
Source: BMC Genomics. 2024 May 31;25:543. doi: 10.1186/s12864-024-10360-9 (PMC11143658; doi:10.1186/s12864-024-10360-9)

**Supporting Data:**

***MIR396-GRF/GIF* enhances in planta shoot regeneration of *Dendrobium catenatum***

Zhenyu Yang^1,2,3^#, Meili Zhao^1,2^#, Xiaojie Zhang^1,2,4^, Lili Gu^4^, Jian Li^1,2^, Feng Ming^3^*, Meina Wang^1,2^*, Zhicai Wang^1,2^*

1 Shenzhen Key Laboratory for Orchid Conservation and Utilization, the National Orchid Conservation Center of China and the Orchid Conservation & Research Center of Shenzhen, Shenzhen 518114, China

2 Key Laboratory of National Forestry and Grassland Administration for Orchid Conservation and Utilization, the National Orchid Conservation Center of China and the Orchid Conservation & Research Center of Shenzhen, Shenzhen 518114, China

3 Shanghai Key Laboratory of Plant Molecular Sciences, College of Life Sciences, Shanghai Normal University, Shanghai 200234, China

4 Xinjiang Key Laboratory of Grassland Resources and Ecology, College of Grassland Sciences, Xinjiang Agricultural University, Urumqi 830052, China

#The authors contributed equally to this work

***Corresponding Authors**

Email: wzcxjnu.5525@163.com (Z.W.); 328365210@qq.com (M.W.); fming@fudan.edu.cn (F.M.)

**Table S1**. Primers used in this study

| Vector construction primers | | |
| --- | --- | --- |
| *TaGRF4-GIF1* | Forward | 5'-ATGGCGATGCCGTATGC-3' |
|  | Reverse | 5'-GCTTCCTTCCTCCTCGGTG-3' |
| *DcGRF4-GIF1* | Forward | 5'-ATGAACAGTACGGCGGCAG-3' |
|  | Reverse | 5'-GTTACCTTCTGCTTCTGAACCTTTC-3' |
| *MIM396* | Forward | 5'-GGTACCGGATCCGCCGTAGC-3' |
|  | Reverse | 5'-GTCGACGCCGGCTACGGCAG-3' |
| qRT-PCR primers | | |
| *qTa/mTaGRF4-GIF1* | Forward | 5'-TCGATCTCGATACCCATGACG-3' |
|  | Reverse | 5'-GTGGTGGCAGTGGTAGAGGAA-3' |
| *qDc/mDcGRF4-GIF1* | Forward | 5'-CGTAACTCTTTCTCTACCACACAGCT-3' |
|  | Reverse | 5'-CCTGGTTCGGAGAAGCGTAA-3 |
| *qMIM396* | Forward | 5'-CGGCAGGTCTTCTCCCTCTA-3' |
|  | Reverse | 5'-TACGGCAGATCTTCCATCAAAAT-3' |
| *qhptII* | Forward | 5'-CTATTTCTTTGCCCTCGGACGAG-3' |
|  | Reverse | 5'-CAATGTCCTGACGGACAATGGC-3' |
| *qNbActin7* | Forward | 5'-CGTCTGTGATAATGGGACTGGA-3' |
|  | Reverse | 5'-CATCCCAGTTGCTAACAATACCAT-3' |
| *qDcActin* | Forward | 5'-GAAGCCCAGTCCAAAAGAGGTATCC-3' |
|  | Reverse | 5'-ACATGGCAGGCACATTGAAAGTCTC-3' |
| *qNtActin7* | Forward | 5'-CGTCTGTGATAATGGGACTGGA-3' |
|  | Reverse | 5'-CATCCCAGTTGCTAACAATACCAT-3' |

***MIM396* sequence**: **^1^**

GGTACCGGATCCGCCGTAGCCGGCAGGTCTTCTCCCTCTAGAAATTGTTCAAGAGCTCAGCTGTGGAAAGCTTCGGTTTTTCTCTTTGGAATGTTCAAGAGCGCAGCTGTGGAATTTTTCAATTTTTTTGGTTGGAATGTTCAAGAGCTTAGCTGTGGAATTTTGATGGAAGATCTGCCGTAGCCGGCGTCGAC

**References**

(1) Soto-Suárez, M.; Baldrich, P.; Weigel, D.; Rubio-Somoza, I.; San Segundo, B. The *Arabidopsis* *miR396* mediates pathogen-associated molecular pattern-triggered immune responses against fungal pathogens. *Sci. Rep.* **2017**, *7* (1), 44898.

**Figure S1**. Uncropped gel images correspond to Figure 4B-H.

Figure 4B: *mDcGRF4-GIF1*


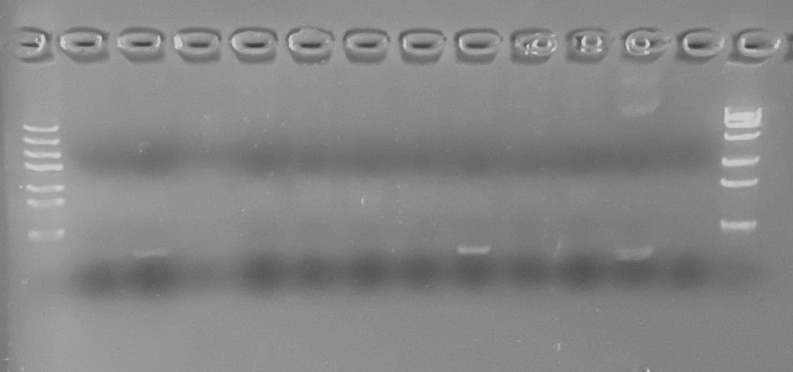


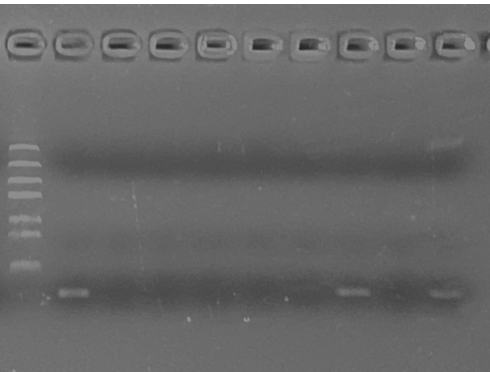


Figure 4C: *TaGRF4-GIF1*

*
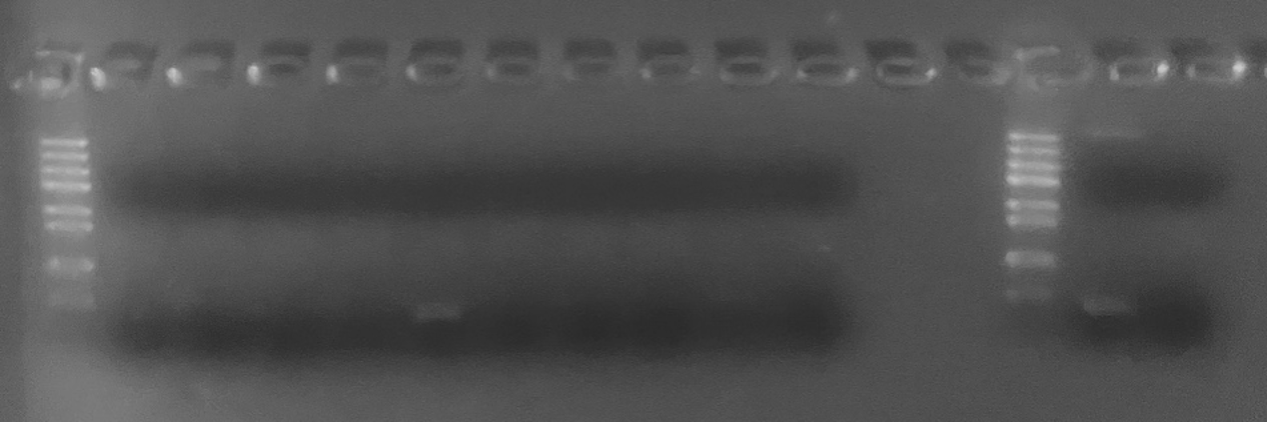
*

Figure 4D: *MIM396*

*
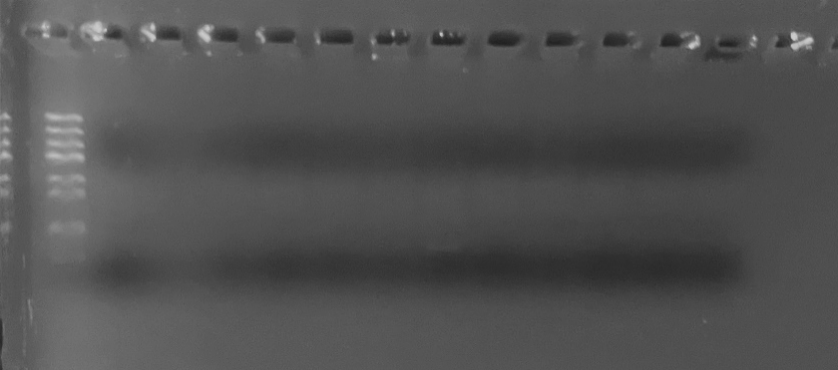
*

Figure 4E: *DcGRF4-GIF1*

*
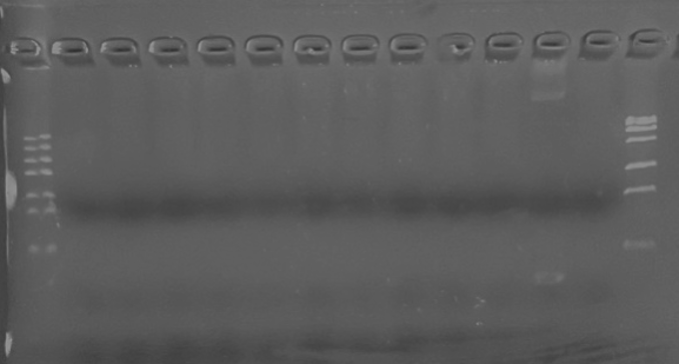
*

Figure 4F: *mTaGRF4-GIF1*


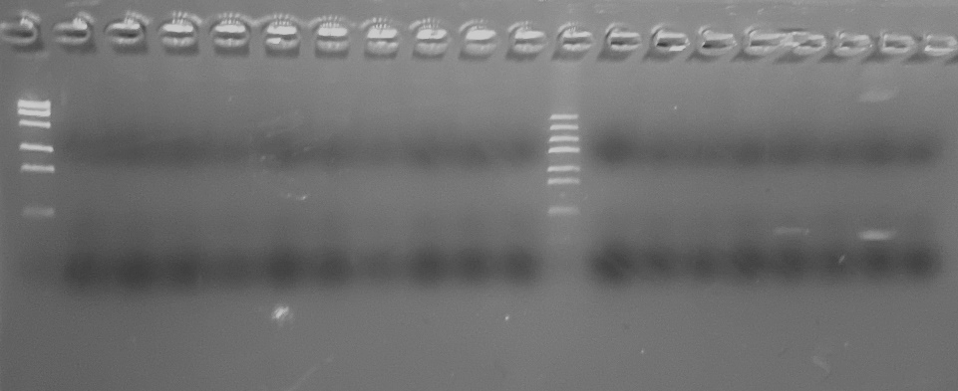


*
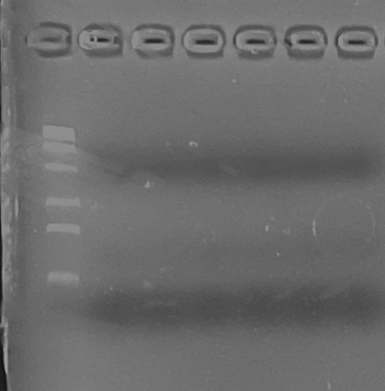
*

Figure 4G: EV

*
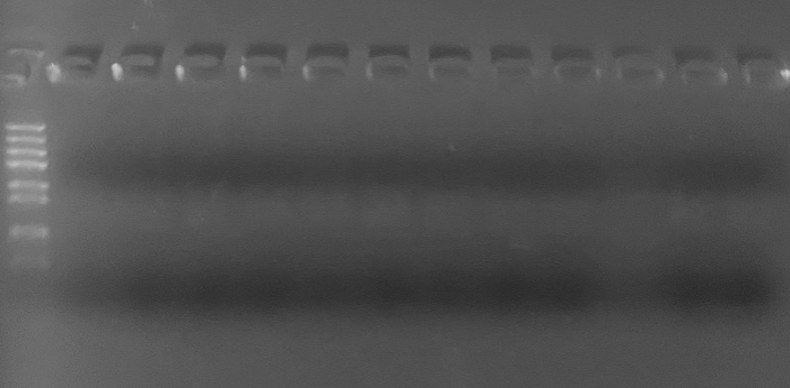
*

*
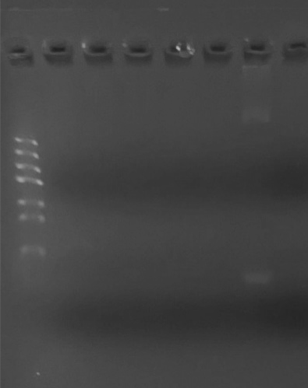
*

Figure 4H: *MIM396*


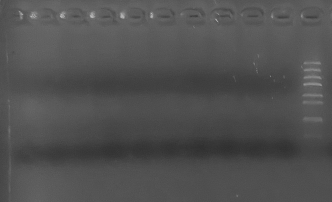


**Figure S2**. Uncropped gel images correspond to Figure 5G.

*Actin*:


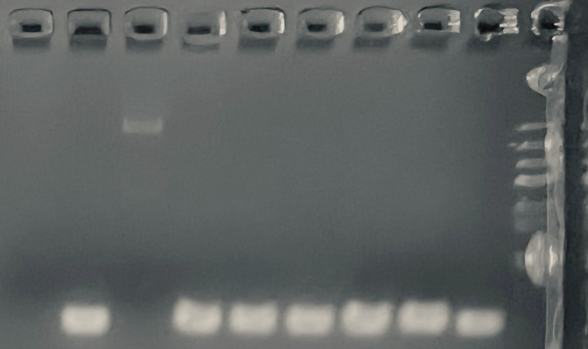


*hptII*:


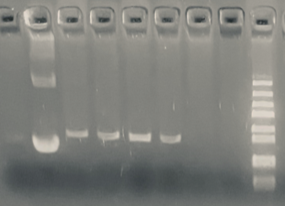


*MIM396*:


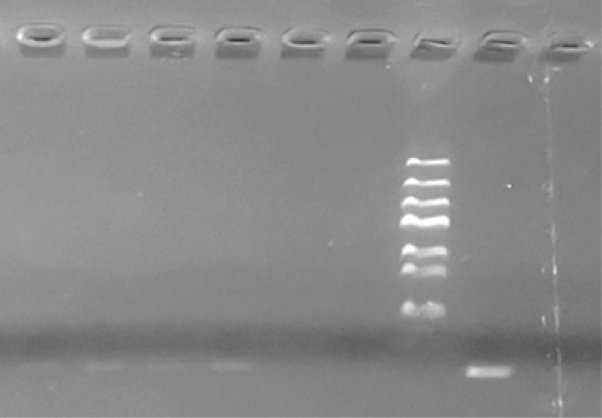


*nptII*:


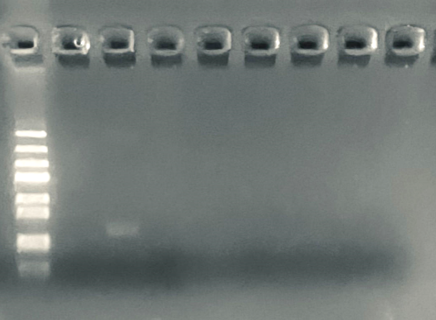

Supplement: Supplementary file 1 — Supplementary Material 1 [file 12864_2024_10360_MOESM1_ESM.docx]
